# Inter-Seasonal Influenza is Characterized by Extended Virus Transmission and Persistence

**DOI:** 10.1371/journal.ppat.1004991

**Published:** 2015-06-24

**Authors:** Zoe Patterson Ross, Naomi Komadina, Yi-Mo Deng, Natalie Spirason, Heath A. Kelly, Sheena G. Sullivan, Ian G. Barr, Edward C. Holmes

**Affiliations:** 1 Marie Bashir Institute for Infectious Diseases and Biosecurity, Charles Perkins Centre, School of Biological Sciences, and Sydney Medical School, The University of Sydney, Sydney, New South Wales, Australia; 2 World Health Organisation Collaborating Centre for Reference and Research on Influenza, Peter Doherty Institute, Melbourne, Victoria, Australia; 3 VIDRL, Peter Doherty Institute, Melbourne, Victoria, Australia; 4 National Centre for Epidemiology and Population Health, The Australian National University, Canberra, Australian Capital Territory, Australia; Plymouth University, UNITED KINGDOM

## Abstract

The factors that determine the characteristic seasonality of influenza remain enigmatic. Current models predict that occurrences of influenza outside the normal surveillance season within a temperate region largely reflect the importation of viruses from the alternate hemisphere or from equatorial regions in Asia. To help reveal the drivers of seasonality we investigated the origins and evolution of influenza viruses sampled during inter-seasonal periods in Australia. To this end we conducted an expansive phylogenetic analysis of 9912, 3804, and 3941 hemagglutinnin (HA) sequences from influenza A/H1N1pdm, A/H3N2, and B, respectively, collected globally during the period 2009-2014. Of the 1475 viruses sampled from Australia, 396 (26.8% of Australian, or 2.2% of global set) were sampled outside the monitored temperate influenza surveillance season (1 May – 31 October). Notably, rather than simply reflecting short-lived importations of virus from global localities with higher influenza prevalence, we documented a variety of more complex inter-seasonal transmission patterns including “stragglers” from the preceding season and “heralds” of the forthcoming season, and which included viruses sampled from clearly temperate regions within Australia. We also provide evidence for the persistence of influenza B virus between epidemic seasons, in which transmission of a viral lineage begins in one season and continues throughout the inter-seasonal period into the following season. Strikingly, a disproportionately high number of inter-seasonal influenza transmission events occurred in tropical and subtropical regions of Australia, providing further evidence that climate plays an important role in shaping patterns of influenza seasonality.

## Author Summary

Human influenza virus commonly causes disease in the winter months of temperate countries, but exhibits more complex patterns in tropical localities. Most studies of this complex seasonality have only considered viruses sampled within the “normal” influenza season. To help reveal the drivers of influenza seasonality we utilized viruses sampled outside of the normal influenza season, focusing on Australia which is characterized by a wide range of climates. Using a phylogenetic approach we revealed more complex patterns of influenza transmission than previously anticipated, particularly that the virus is able to transmit for extended periods and even persist locally within Australia throughout the virus “off-season”. In addition, we found that inter-seasonal influenza was more frequent in tropical and sub-tropical than temperate regions, adding weight to theories that climate likely plays an important role in influenza seasonality.

## Introduction

Human influenza virus is characterized by a marked seasonality in temperate regions, where the virus exhibits a distinct annual peak in epidemic activity during the winter months [1]. However, in subtropical and tropical regions transmission patterns are often less clearly defined [2]. Indeed, the epidemiological dynamics of influenza in tropical regions are more commonly characterized by continuous low rates of disease throughout the year, with semi-annual epidemics in some regions [3–5].

Two main categories of factor appear to drive these complex patterns of influenza seasonality [1,6,7]: those associated with changes in host behavior, perhaps driven by differing environmental conditions, and changes in the physical properties of the virus (or host) that reflect large-scale environmental changes. Examples of potential behavioral drivers of influenza seasonality include patterns of school attendance or crowding indoors during inclement weather [8–10], while potential environmental factors may include changes in humidity and temperature that affect virion stability and hence virus survival [11–13].

Understanding the patterns of viral activity in different climatic regions and at different times is central to revealing the determinants of influenza seasonality. However, those studies undertaken to date have generally focused on patterns of virus transmission within the defined influenza season in temperate regions [14]. Indeed, there is a marked absence of studies of viral transmission and evolution outside of the usual time-scale of influenza seasons (with an epidemiological study of the inter-seasonal 2010/2011 period in Australia an exception [15]), even though these may provide an important perspective on influenza seasonality.

Inter-seasonal influenza is generally thought to involve the importation of an influenza virus from a locality either in the alternate hemisphere where the influenza season is current [6], or from the tropics where low levels of virus may circulate year-round [2,3], particularly the densely populated regions of East and South-East Asia [16]. However, once in a local inter-seasonal period, it is expected that migrant viruses will not be able to achieve onward transmission in the population due to unfavorable behavioral or environmental conditions. As a consequence, inter-seasonal occurrences of influenza are generally assumed to represent sporadic importations that do not play a major role in global viral transmission dynamics [14,17].

Theoretically, viral lineages may also persist over the inter-seasonal period in specific populations. To date, however, there is limited evidence for local multi-seasonal viral persistence. For example, phylogenetic analysis has provided evidence for the persistence of lineages of pandemic influenza A/H1N1pdm in West Africa (although not within a single country) [18] and Vietnam [19], as well as highly pathogenic A/H5N1 avian influenza viruses, again in South-East Asia and Africa [20]. However, such instances are rare, with little evidence for the persistence of A/H3N2 influenza virus [16–17,21–22], even in cases where there is evidence for transmission into the summer months [23], although it is possible that this in part reflects poor or biased sampling.

**Fig 1:**
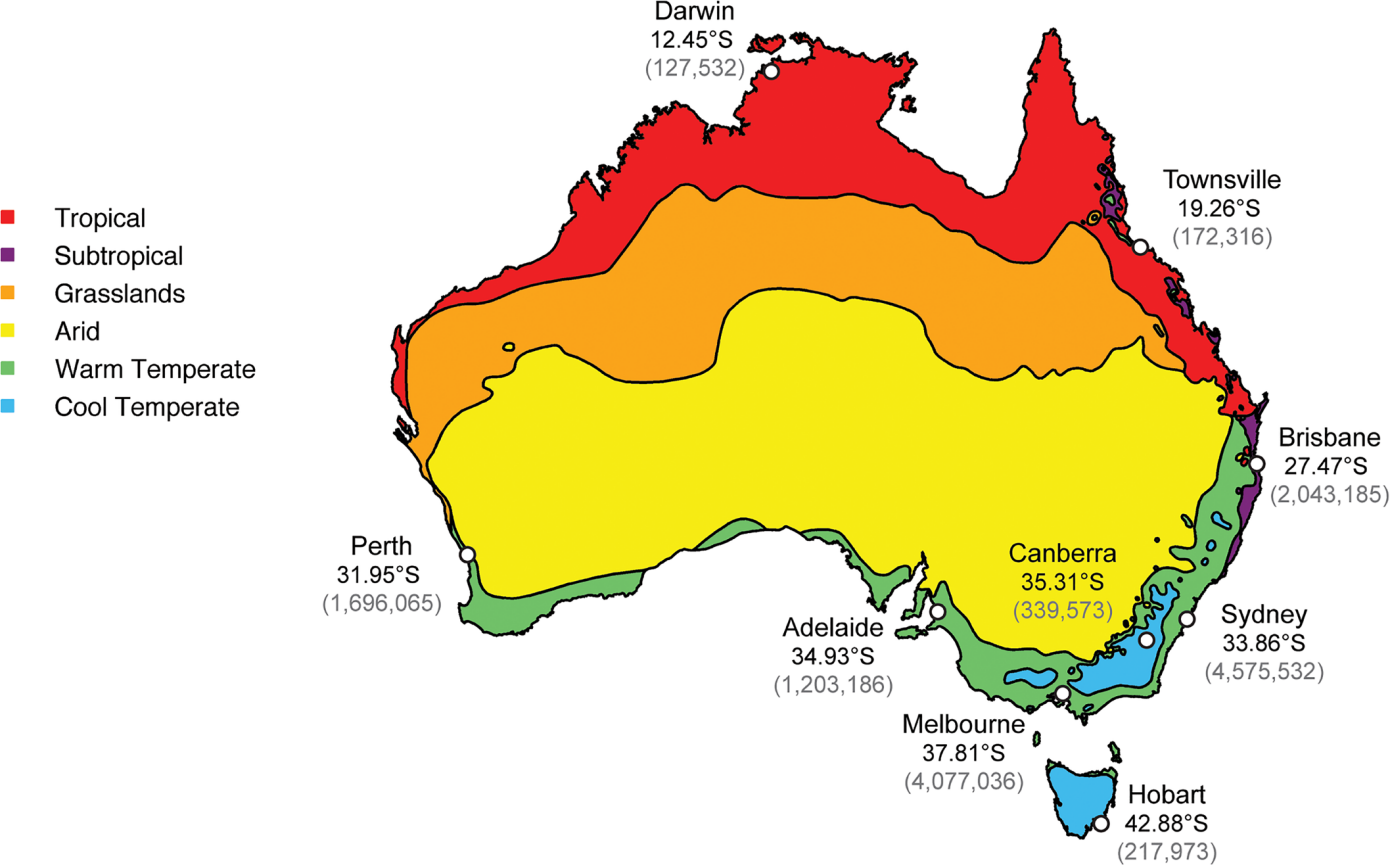
Climate zones and the location of the most populous cities within Australia. The map was constructed using freely available data from the Bureau of Meteorology’s website (www.bom.gov.au) and utilizes their temperature/humidity classification system [25]. Population data was from the Australian Bureau of Statistics [26]. Latitude figures are freely available on Google Maps.

Herein we focus on the patterns and dynamics of inter-seasonal influenza in a single country–Australia–and ask how viruses sampled during this period are related to those sampled on a global scale. Previous studies of influenza in Australia have considered influenza circulation at a regional level with a focus on seasonal influenza, with relatively little consideration of inter-seasonal activity [15,24]. However, the availability of both epidemiological and sequence data makes Australia an ideal study site for a wider study of influenza seasonality, allowing us to reveal the origin and spread of influenza viruses during the inter-seasonal period. In light of the possible role played by climate in driving influenza seasonality [6], we also sought to identify potential links between climatic factors and the occurrence of inter-seasonal influenza, a task made possible by the broad range of climatic zones in Australia (Fig 1).

Accordingly, we performed an expansive phylogenetic analysis of a global data set of hemagglutinin (HA) glycoprotein coding sequences from over 17,500 influenza A/H1N1pdm, A/H3N2 and B virus sequences sampled over a five-year period from 2009–2014, of which 396 (2.2%) were sampled during the inter-seasonal period in Australia. In particular, we sought to determine whether these inter-seasonal viruses were seeded from global sources (i.e. were imports) or were part of a lineage able to transmit for extended periods within Australia, including temperate regions of the country. We focused on 2009–2014 as laboratory testing of samples increased following the 2009 human A/H1N1 pandemic (H1N1pdm), yielding a relatively rich data set.

## Methods

### Virus sampling and HA gene sequencing

Full-length HA sequences were collected both during the temperate seasonal and inter-seasonal period in Australia during 1 Jan 2009–31 Jan 2014. The seasonal model for influenza is perhaps less strictly descriptive of the shape and peak of influenza occurrence in Australia compared to wholly temperate countries in the northern hemisphere, although still of importance in predicting when the peak of influenza incidence will occur (usually late July/August) and when to implement vaccination campaigns. The influenza season in temperate Australia (where the bulk of the population resides and which contains the main transportation hubs) was therefore defined as occurring between 1 May and 31 October, particularly as surveillance activities focus on this as the peak time for influenza viral activity [24]. Consequently, inter-seasonal events were classified here as those that occurred between 1 November and 30 April. The more complex pattern in the less populous tropical northern Australia is then discussed in relation to these temperate seasonal boundaries. Respiratory samples or influenza virus isolates were collected from National Influenza Centres and laboratories across all eight Australian states and territories. Clinical samples and isolates were passaged in MDCK cells (ATCC CCL-34) and resultant viruses had their HA genes sequenced as previously described [27]. Briefly, virus samples were cultured in Madin-Darby canine kidney (MDCK ATCC CCL-34) cells as previously described [27] and RNA was extracted from the virus isolates using QIAamp Viral RNA Mini Kit (Qiagen) according to the manufacturer’s instructions. RT-PCR using MyTaq One-Step RT-PCR kit (Bioline) with subtype specific HA primers (primer sequences available upon request). RT-PCR products were purified by ExoSAP-IT (GE Healthcare) and sequenced with Big Dye Terminator Reaction Mix (Applied Biosystems) and run on ABI 3500 XL following the manufacturer’s instructions. All sequences generated here have been deposited at the Global Initiative on Sharing all Influenza Data (GISAID; http://platform.gisaid.org/) database and assigned accession numbers as listed in S1 Table. These data comprised 148 pandemic A/H1N1 (A/H1N1pdm) sequences, 66 A/H3N2 sequences, and 39 influenza B sequences.

### Evolutionary analysis

The sequences generated here were combined with 456 A/H1N1pdm, 274 A/H3N2 and 276 influenza B HA sequences from Australia sampled during the same time period (2009–2014, including both seasonal and inter-seasonal data) and downloaded from GISAID. To place the Australian sequences in the context of global influenza virus genetic diversity, data sets of full-length HA sequences from each influenza virus subtype (A/H1N1pdm, A/H3N2, and B) sampled worldwide during 2009–2014 were compiled using GISAID and GenBank (http://www.ncbi.nlm.nih.gov/genbank/). Only sequences for which the collection date was known were included. Duplicate sequences (i.e. collected from the same location on the same date and which appear to differ only in passage history) were excluded to improve computational tractability. Consequently, final HA data sets of 9,912 A/H1N1pdm sequences, 3,804 A/H3N2 sequences and 3,941 influenza B virus sequences, with total numbers of Australian seasonal/inter-seasonal sequences of 546/149 for A/H1N1pdm, 244/108 for A/H3N2, and 292/139 for influenza B, were utilized for phylogenetic analysis.

Sequences of each influenza subtype were aligned separately using the MAAFT program (v7.017; [28]) available through Geneious (v7.1.3; http://www.geneious.com/), followed by manual adjustment. To obtain an initial view of the phylogenetic relationships of each of the three data sets, phylogenetic trees were inferred using the maximum likelihood (ML) method available in RAxML [29]. This returned the best tree from 20 replicates inferred using the general time-reversible (GTR) substitution model with a gamma (Γ) distribution of among-site rate variation. All parameter values were estimated from the empirical data (available upon request).

To assess the reliability of key nodes (i.e. those pertinent to inter-seasonal influenza virus transmission in Australia), a second ML phylogenetic analysis was undertaken on clades comprising largely Australian samples, this time incorporating bootstrap resampling. Accordingly, clades were selected for further analysis if they contained isolates spanning a date range outside of the influenza surveillance season. We then inferred 1000 replicate ML trees in PhyML [30] using the GTR+Γ substitution model described above. The resulting phylogenies were visualized using FigTree (v1.4.1; available at: http://tree.bio.ed.ac.uk/software/figtree/). Only bootstrap values >70% were considered as statistically robust [31].

### Classification of epidemiological events in Australia

**Fig 2:**
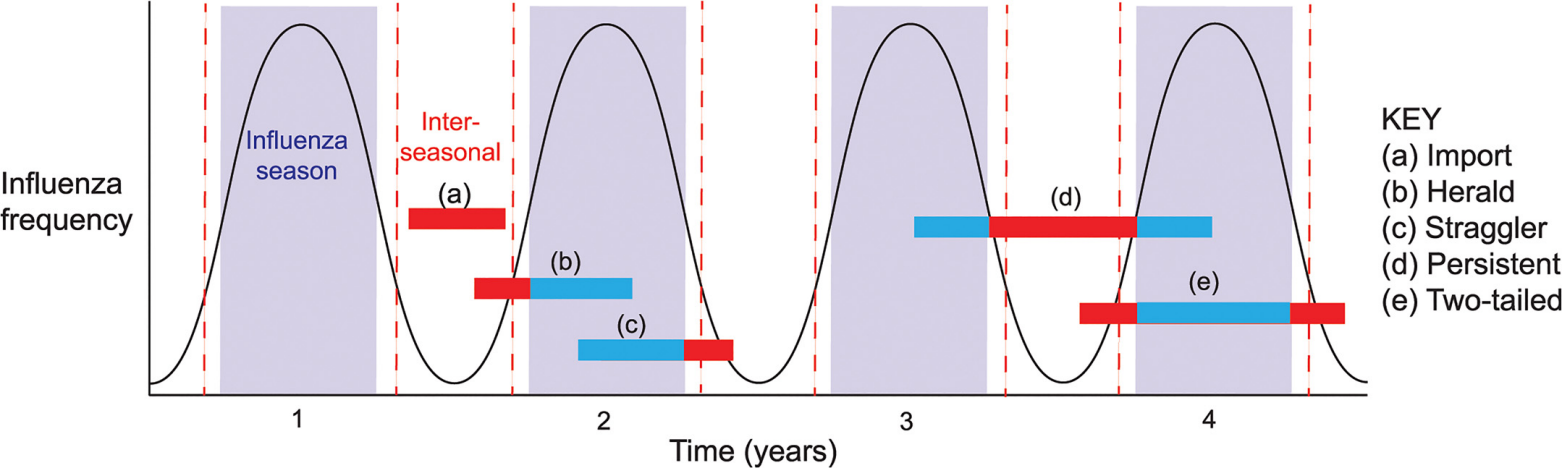
Patterns (event types) of inter-seasonal influenza transmission. The black line represents the theoretical periodicity of influenza in a typical seasonal pattern, horizontal bars represent lengths of transmission events and are colored blue when falling inside the season and red when in the inter-seasonal period.

To determine the epidemiological context of influenza viruses sampled inter-seasonally in Australia, we classified them into five categories (denoted a-e) based on their phylogenetic position within the trees described above (schematic epidemiological patterns are shown in Fig 2; see below). We assumed that Australian sequences that cluster together in the context of the global background denoted transmission events that most likely occurred within Australia, although every event type (category) necessarily began with an importation into Australia. Finally, geographic location data (to the nearest town/city) was available for the majority of the inter-seasonal sequences, and was used as a proxy for location of viral infection. The five categories of epidemiological events were:

- *Import*: If a single Australian isolate fell in a clade containing only globally sampled sequences, with no close phylogenetic relationship to other Australian sequences, then it was considered to result from an importation causing only limited onward transmission in Australia. Although most imports were only sampled once, and hence may have experienced only limited transmission in Australia, some comprised lineages of multiple sequences such that they were clearly transmitted for extended periods inter-seasonally. Although, as noted above, all transmission events studied here began with an importation event, this category as defined here only considers transmissions within the inter-seasonal period.
- *Herald*: If an Australian isolate was closely related to global sequences, but clustered with and preceded a number of other Australian isolates from the forthcoming influenza season, it was considered to be part of a “herald” event. Hence, we assumed that herald lineages persisted from within the inter-seasonal period into the next influenza season following an international importation.
- *Straggler*: If an inter-seasonal Australian isolate exhibited a close phylogenetic relationship to Australian isolates from the preceding influenza season, but did not persist into the following season, then it was considered to be part of a “straggler” event.
- *Persistent*: Isolates were classified as being part of a persistence event if they were part of a cluster of Australian isolates that occurred inter-seasonally, with only a limited number of related global sequences, and which were related to those from *both* the preceding and subsequent seasons such that they are indicative of continual local inter-seasonal transmission.
- *Two-tailed*: Finally, viral lineages that transmitted before, during and after the normal influenza season, such that they contain herald, seasonal and straggler components but which were not classified as persistent as they were not maintained throughout the inter-seasonal period, were termed “two-tailed” lineages.

To explore the effect of climate on influenza seasonality in Australia, we used the Australian Bureau of Meteorology’s climate classifications. These recognize three commonly used methods for classifying the climate of mainland Australia: (i) temperature/humidity maps, compiled from data collected nationally over the period 1961–1990 and comprising six key zones; (ii) modified Köppen maps, which again show six major groups, but with 27 sub-classifications; and (iii) seasonal rainfall levels, which again identify six major climate groups [25]. The six key groups identified by the temperature/humidity maps were used as the basis of climate classification for cities and towns in this study (Fig 1). The locations of interest here were classified consistently in all three methods, with the only differences reflecting levels of rainfall and summer temperatures. For instance, Townsville and Darwin are both classified as “tropical” in the Köppen system and as having “hot, humid summers” in the temperature/humidity zones, whereas Sydney and Canberra are both classified as “temperate” in the Köppen system, but their summers are differentially classified as “warm” and “mild/warm” in the temperature/humidity zones [25].

## Results

### Inter-seasonal transmission events in Australia

Our phylogenetic analysis of 17,657 HA gene sequences sampled globally during 2009–2014 (S1–S3 Data) revealed that multiple lineages of each influenza virus established extended transmission chains in both temperate and tropical Australia during inter-seasonal periods, including persistence in the case of influenza B viruses (Table 1).

**Table 1:** Number of sequences in each epidemiological event type by climatic zone (temperate, sub-tropical, or tropical) in Australia.

	Temperate			Subtropical			Tropical				
	Number of sequences per event type	% sequences per event type per subtype	% of total number of sequences per subtype	Number of sequences per event type	% sequences per event type per subtype	% of total number of sequences per subtype	Number of sequences per event type	% sequences per event type per subtype	% of total number of sequences per subtype	Total number of sequences per event type	% of sequences per event type
**A/H1N1pdm**										149	
Import	39	44.83	26.18	23	26.44	15.43	25	28.74	16.78	87	58.39
Herald	4	22.22	2.68	10	55.56	6.71	4	22.22	2.68	18	12.08
Straggler	13	41.94	8.72	7	22.58	5.37	11	35.48	7.38	31	20.81
Persistent	0	0.00	0.00	0	0.00	0.00	0	0.00	0.00	0	0.00
Two-Tailed	3	23.08	2.01	9	69.23	6.04	1	7.69	0.67	13	8.72
**A/H3N2**										108	
Import	37	67.27	34.26	12	21.82	11.11	6	10.91	5.56	55	50.93
Herald	6	35.29	5.56	5	29.41	4.63	6	35.29	5.56	17	15.74
Straggler	15	41.67	13.89	3	8.33	2.78	18	50.00	16.67	36	33.33
Persistent	0	0.00	0.00	0	0.00	0.00	0	0.00	0.00	0	0.00
Two-Tailed	0	0.00	0.00	0	0.00	0.00	0	0.00	0.00	0	0.00
**B**										139	
Import	31	59.62	22.30	13	25.00	9.35	8	15.38	5.76	52	37.41
Herald	15	83.33	10.79	2	11.11	1.43	1	5.56	0.67	18	12.95
Straggler	19	48.72	13.67	7	17.95	5.04	13	33.33	9.35	39	28.06
Persistent	6	35.29	4.32	9	52.94	6.47	2	11.76	1.44	17	12.23
Two-tailed	9	69.23	6.47	1	7.69	0.72	3	23.08	2.16	13	9.35
Total Sequences	197			101			98				

**Fig 3:**
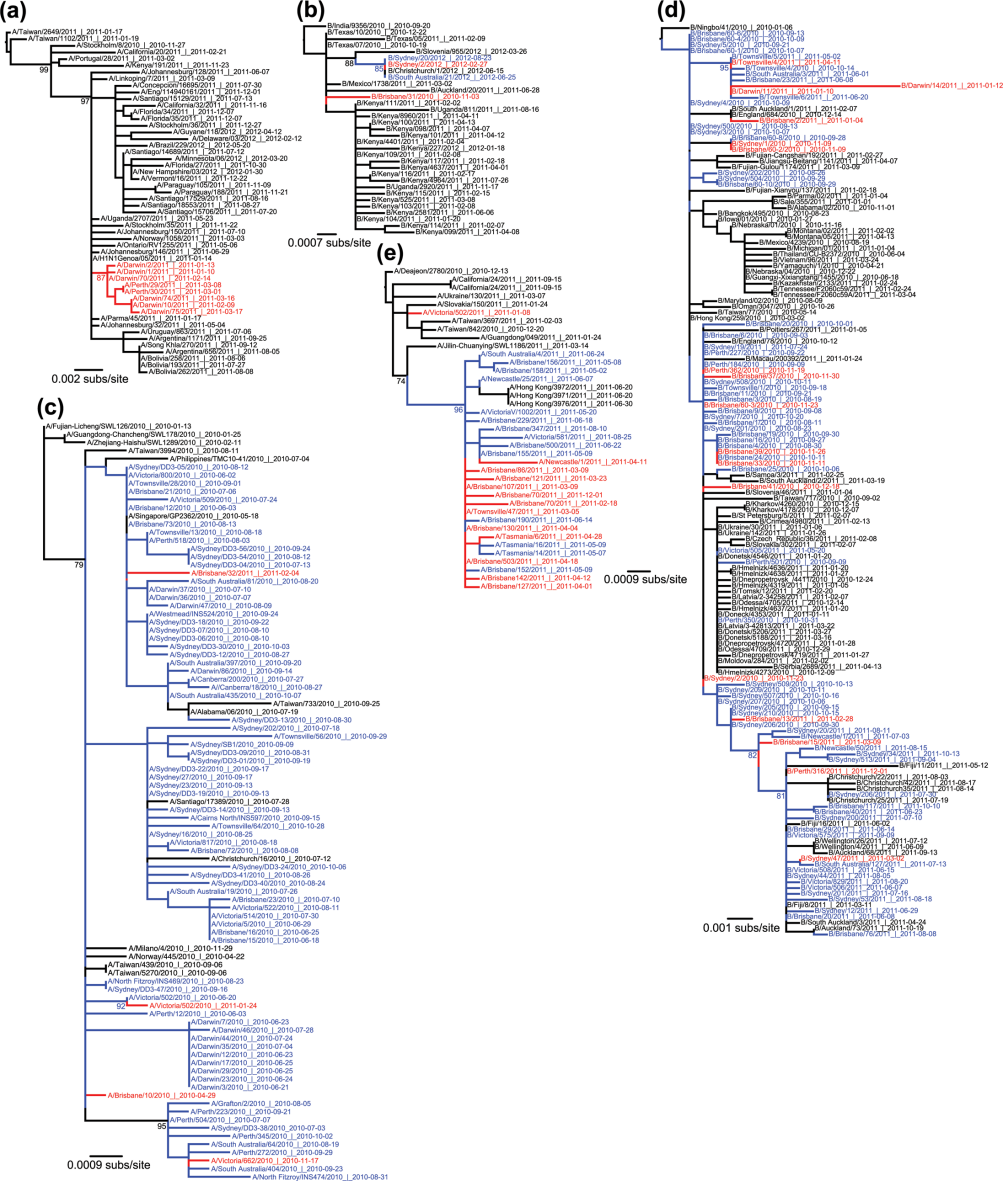
Phylogenetic trees showing a real data example of each event type. Australian inter-seasonal sequences are shown in red, seasonal sequences are shown in blue, and global sequences are shown in black. Bootstrap support values (>70%) are shown for key nodes. (**a**) Importation event within influenza A/H1N1pdm. This imported lineage transmitted for at least two months during the 2010/2011 inter-seasonal period within Australia (earliest sample collected on 13 Jan 2011 and latest on 18 March 2011, both from Darwin), and spread to a temperate region (Perth). (**b**) Herald event within influenza B virus, with virus Sydney/2/2012 isolated on 27 February 2012 and then extending into the following influenza season in both Australia (Sydney and South Australia) and New Zealand. (**c**) Straggler event within influenza A/H1N1pdm, with viruses transmitting within the 2010 season into the 2010–2011 inter-seasonal period. Although the strongest evidence for this involves A/Victoria/662/2010 isolated on 17 November 2010 (bottom clade), a similar pattern may occur with A/Brisbane/32/2011 sampled on 4 February 2011 although with weak bootstrap support (top clade). (**d**) Persistent lineage of influenza B virus transmitting within the 2010 season, throughout the 2010/2011 inter-seasonal period, and into the 2011 season (i.e. for a total of 14 months). (**e**) A two-tailed A/H1N1pdm lineage transmitting from 18 February 2011 (2010/2011 inter-seasonal period) to 01 December 2011 (2011/2012 inter-seasonal period). All trees were rooted using the earliest non-Australian sequence shown to be an outgroup in the expansive phylogenetic trees shown in S1 Fig. The date of sampling is shown in the sequence label in the format year-month-day.

To examine the patterns of inter-seasonal transmission and evolution in more detail, we performed focused phylogenetic analyses of each possible transmission type (Table 1 and Fig 3). All five types of inter-seasonal events–import, herald, straggler, persistent, two-tailed–were observed. Of these, importation was the most common and represented 58.4% of all inter-seasonal sequences in A/H1N1pdm, 50.9% in A/H3N2, and 37.4% in influenza B virus (Table 1). Frequencies for each event type seemingly differed between influenza A subtypes A/H1N1pdm and A/H3N2, and between influenza A and B (Table 1). However, because of relatively limited and non-systematic sampling available here it was unclear whether this represented a fundamental difference in epidemiological dynamics between subtypes [17] or ascertainment bias.

Although influenza transmission events have previously been documented outside of seasonal boundaries [23,32], the inter-seasonal events documented here were lengthier than expected, included those of potential evolutionary importance such as local persistence, and sometimes involved viruses sampled from both tropical and temperate regions within Australia. According to our phylogenetic analysis, 70 Australian viruses appeared in herald events, denoting viruses that appeared prior to the local influenza season and continued to transmit into the full season, while 115 could be classified as stragglers that continued to transmit beyond the end of the season.

Notably, although we observed imports, stragglers, and heralds within Australia for both A/H3N2 and A/H1N1pdm, no evidence for inter-seasonal persistence was observed in either virus. Rather, the best evidence for persistence came from a clade of influenza B virus (Victoria lineage) that included 92 Australian isolates, of which 17 were collected during the summer of 2010/2011 (Fig 3D). This clade appeared to persist locally within the 2010 season (earliest date isolated: 11 August 2010, sequence B/Victoria/503/2010) and through the 2011 season (latest date isolated: 13 October 2011, B/Sydney/34/2011), with most major branches showing bootstrap support values over 70%. Importantly, eight isolates from this event, spanning an eight-month period linking the 2010 and 2011 seasons, clustered strongly together to the exclusion of non-Australian sequences (94% bootstrap support). The earliest virus within this group was isolated in Townsville (in tropical northern Australia) at the end of the 2010 season (14 October 2010). Three inter-seasonal isolates followed, two from Darwin (also located in tropical northern Australia) in January 2011 and another from Townsville in April of that year. In the 2011 season, the earliest sequence was again from Townsville (02 May 2011), followed by isolations in South Australia (a temperate region; 01 June 2011), Brisbane (subtropical; 08 June 2011) and Townsville (20 June 2011). Earlier sequences, which appeared to be related to this cluster albeit with weaker bootstrap support, were all from 2010, and came from Sydney (temperate), and Brisbane. Despite incomplete global sampling, clearly the most parsimonious explanation with the phylogenetic data in hand is that the influenza B viruses in question have transmitted locally for the full duration of the Australian summer, and spread within both tropical and temperate regions.

**Fig 4:**
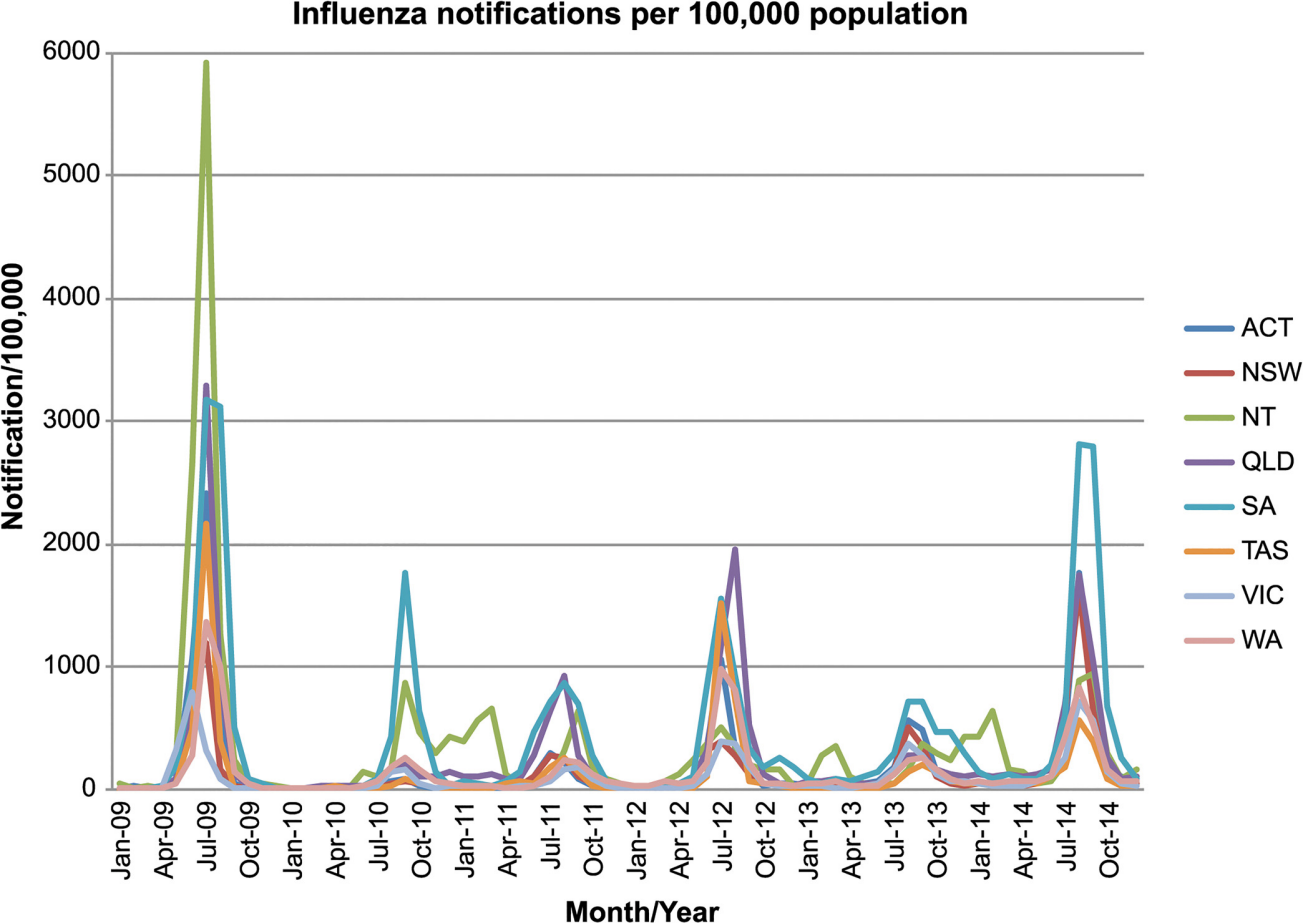
NNDSS influenza notifications in Australia. Laboratory-confirmed influenza notifications throughout the seasonal and inter-seasonal period in Australia. The plot shows laboratory-confirmed diagnoses per 100,000 population from 2009–2014, with states and territories displayed separately. Data on laboratory confirmed cases of influenza in Australia was obtained from the Australian National Notifiable Diseases Surveillance Systems (NNDSS) website (http://www9.health.gov.au/cda/source/cda-index.cfm).

The 2010/2011, 2012/2013 and 2013/2014 inter-seasonal periods in Australia were characterized by increased numbers of laboratory-confirmed notifications of influenza to the National Notifiable Diseases Surveillance System (NNDSS) (Fig 4). It was previously concluded that the high number of notifications between the 2010 and 2011 seasons likely reflected a genuine increase in disease, magnified by increased laboratory testing of samples following the 2009 H1N1 pandemic, and that this may have been a normal fluctuation in levels of inter-seasonal influenza [15]. Notably, although there was no obvious association between the number of influenza notifications and the types of inter-seasonal transmission events that occurred, our phylogenetic analysis did reveal the presence of a persistent lineage of influenza B virus and two-tailed seasonal lineages of influenza A/H1N1 during the 2010/2011 inter-seasonal period, as well as frequent other inter-seasonal transmission events.

### Geographical patterns of inter-seasonal influenza

The availability of geographical data enabled us to determine whether there was an association between the inter-seasonal transmission and climatic zone within Australia (Table 1). Although the majority of the Australian population resides in temperate regions, our analysis is striking in that the proportions for inter-seasonal influenza were strongly skewed towards sub-tropical and tropical zones, with a disproportionate number of inter-seasonal events falling within Australia’s tropical and subtropical regions (98 and 101 sequences, respectively), as opposed to the temperate regions (197 sequences) (Table 1). Accordingly, the ratio of inter-seasonal isolates sampled in ‘temperate: subtropical: tropical’ zones is ‘2:1:1’, while the size of the population in each of these zones is in the ratio 43:7:1 [26]. Although there are likely biases in those samples selected for sequencing, these would not necessarily result in an increase in apparent inter-seasonal transmission. Indeed, low levels of sampling are likely to under-estimate the true occurrence of inter-seasonal influenza transmission, such that these results err on the conservative side. Accordingly, this result, coupled with the appearance of persistent virus transmission events centering on tropical Darwin and Townsville, suggests that there may be a link between viral persistence and climate. In particular, while the seasonal portions of these extended transmission chains tend to occur in temperate regions, the inter-seasonal transmission period appears to be more frequent in hot and humid localities, also suggesting that there is a high degree of connectivity between these regions.

## Discussion

Our expansive phylogenetic analysis revealed five different types of inter-seasonal influenza transmission in Australia: exotic imports causing (i) limited onward transmission (imports); (ii) onward transmission in the following influenza season (heralds); (iii) stragglers from the preceding season; (iv) persistent viral lineages causing disease throughout the inter-seasonal period and into at least two adjacent seasons; and (v) a “two-tailed” pattern of sustained local influenza transmission that contains both heralds and stragglers.

Virological surveillance data are primarily collected for the early detection and characterization of novel viruses as part of pandemic preparedness, and to inform vaccine strain selection [33]. As a consequence, this analysis, like most in influenza molecular epidemiology, was necessarily biased towards viral infections causing more severe or unusual disease symptoms [34], and which occurred during the influenza season. Despite these intrinsic biases the phylogenetic data presented here provide strong evidence that local chains of extended viral transmission occur within the Australian inter-seasonal period, supporting previous work in this area [15]. Although there was evidence for the persistence of influenza viruses spanning the length of the Australian inter-seasonal period, it was striking that few clades were observed that were exclusively comprised of Australian isolates, again emphasizing the fluidity with which influenza viruses are able to spread [35,36]. Importantly, the close evolutionary relationship of sequences dated earlier and later than those of international sequences implies that they were not the direct ancestors of the Australian viruses, and some viruses may even be exports from Australia. It remains to be seen whether the observations made here hold true in more populous countries also with diverse climates, such as China and the USA.

Most models of global influenza transmission assume that inter-seasonal events are due either to importation from the seasonal epidemic of the northern hemisphere or from virus circulating in tropical regions [2,16,17]. Although we observed many examples of exotic virus importation, our study provided strong evidence that influenza viruses were capable of establishing extended chains of transmission, including persistence, between the normally defined influenza seasons in Australia, and of spreading within clearly temperate regions. While the majority of influenza cases seen during the (winter) influenza season were in the heavily populated temperate cities, such as Sydney (New South Wales), Melbourne (Victoria), and Perth (Western Australia), a key observation of our study was that the occurrence of influenza during the inter-seasonal period occurred disproportionately (although not exclusively) in the less populous tropical and subtropical regions, including Darwin (Northern Territory), Townsville (Queensland), and Brisbane (Queensland). This, in turn, is compatible with the idea that climatic factors, such as humidity, play an important role in determining the epidemiological dynamics of inter-seasonal influenza. Given the smaller number of sequences from tropical/sub-tropical regions, it seems unlikely that the disproportionate representation of inter-seasonal transmission events in tropical and subtropical regions is artefactual. Indeed, it is possible that there are more cases of persistent (and other long-term) virus transmission within Australia than revealed by our analysis, particularly in tropical rural areas where surveillance is less intensive. The increased surveillance of influenza virus outside the normal sampling regions (i.e. urban areas) and normal sampling times (i.e. the influenza season defined for temperate regions) is clearly a priority for the future.

### Regional influenza dynamics

Our study benefits from the fact that we were able to examine the inter-seasonal dynamics of influenza virus in climatically diverse areas. Indeed, our study was noteworthy in that we observed both the inter-seasonal transmission of influenza in temperate regions of Australia (including the most southerly state of Tasmania; Fig 3E), and lengthy inter-seasonal transmission chains centered on sparsely populated tropical and sub-tropical areas, although often closely related to seasonally transmitting viruses from temperate regions. Although this in part clearly reflects aspects of population mobility, including fly-in and fly-out workers, if population mobility were the major factor explaining persistence we might expect to see more frequent persistence in temperate zones where the populations are large, dense, and mobile (including the main national and international transportation hubs). Hence, that we observed greater levels of extended inter-seasonal transmission in the tropical and sub-tropical zones where populations are smaller and less dense is more consistent with a climate-driven effect. However, it is evident that further surveillance of tropical, sub-tropical and other non-temperate climatic zones is needed year-round to ascertain whether persistence can be definitively linked to certain climatic zones.

The climate-driven model of influenza transmission generally considers absolute temperature and relative humidity (RH) to be the driving factors of influenza transmission, although it is has also been suggested that absolute humidity (AH) is a better predictor of influenza virus survival and transmission, or moderates transmission mechanisms [11,37]. We necessarily focused on RH as these are the data provided by the Australian Bureau of Meteorology [11,25], so that the role of AH is difficult to assess here. In tropical Darwin where we observe viral persistence, temperature and RH do not vary widely, with average daily temperatures ranging from only 30 to 33°C throughout the year [25]. The most important difference between the seasonal and inter-seasonal period in Darwin is in the amount of rainfall. From May–October an average monthly rainfall of 14.1 mm is recorded, whereas the equivalent value for November–April is 241.4 mm [25]. Other measures of climatic variability, such as temperature and relative humidity, seem not to differ greatly between the seasonal and inter-seasonal periods. For example, the average maximum/minimum temperature for May–October is 32.2°C/22.07°C, while for November–April it is 33.1°C/25.1°C, with the average 9am/3pm readings for RH at 63.17%/48% from May–October and 74.83%/65.67% for November–April [25].

Experimental studies using guinea pig models have found that the aerosol transmission of influenza virus was blocked or inefficient at 30°C and intermediate-high humidity (50–80% RH) [11,12], such that it was favored in cool and dry conditions. It is therefore unclear how influenza virus transmission occurs in the tropics. It is possible that much transmission occurs indoors, for example mediated by air-conditioning, or that contact transmission is more efficient in warm humid conditions, for instance if droplets of mucus that contain virus desiccate at a lower rate in high humidity. Studies on the relationship between absolute humidity and influenza virus survival and transmission have provided evidence for increased survival and transmission at both low and high absolute humidity, suggesting a potential bimodal relationship [37,38]. Large-scale epidemiological studies have also shown that the association between peaks in influenza activity and climatic variables, such as temperature, humidity and solar radiation, varies with latitude, being strongest at latitudes higher than 25°S, and no significant association between 12.5–25°S [39]. Within our study population, Darwin sits at 12.4°S, Townsville at 19°S, Brisbane at 27°S, while all temperate cities are at higher latitudes (at around 33°S). Accordingly, there should be no significant association between influenza peaks and climatic variables in the Australian tropics. Clearly, further work is needed to determine whether the populations in tropical and sub-tropical areas have high rates of inter-seasonal influenza due to increased host susceptibility, or environmental factors specific to their location, or some combination of these factors.

Previous studies of influenza seasonality [40,41] have described Australia as a “temperate” country, presumably as the most populous cities are located within temperate zones. However, this does not take into account Australia’s large climatic diversity, nor the epidemiology of influenza within populations outside temperate zones which can be more complex than a single seasonal peak. Whatever the classification method utilized, it is important that studies of influenza epidemiology in Australia reflect its climatic complexity, particularly as models of influenza seasonality are being used to inform vaccination strategies within Australia [42,43].

Influenza circulation is clearly complex, involving an interplay between climate, viral movement, population mobility, and aspects of population immunity and susceptibility. We have revealed an unexpectedly important role for inter-seasonal influenza transmission in both tropical and temperate regions. While it is clear that the vast majority of influenza cases occur during the temperate influenza season, the thresholds of these seasons may change with variations in seasonal climatic factors such as temperature and humidity year-to-year. A greater focus on the occurrence and determinants of inter-seasonal influenza may provide data central to determining the key drivers of influenza seasonality.

## Supporting Information

S1 Fig Phylogenetic trees of influenza viruses using the full sequence (global) data sets collected between 2009 and 2014. (**A**) influenza A/H1N1pdm, (**B**) A/H3N2 and (**C**) influenza B virus. Australian inter-seasonal sequences are shown in red, seasonal sequences are in blue, and global background sequences are in black. The trees are shown in a “circular” format for ease of visualization only.
(EPS)

S1 Data Phylogenetic tree (including virus names and collection dates) in nexus format for the entire A/H1N1pdm data set collected globally during 2009–2014. Australian inter-seasonal sequences are shown in red, seasonal sequences are in blue, and global background sequences are in black, with branch lengths scaled according to the number of nucleotide substitutions per site.
(TXT)

S2 Data Phylogenetic tree in nexus format for the entire A/H3N2 data set collected globally during 2009–2014. Australian inter-seasonal sequences are shown in red, seasonal sequences are in blue, and global background sequences are in black, with branch lengths scaled according to the number of nucleotide substitutions per site.
(TXT)

S3 Data Phylogenetic tree in nexus format for the entire influenza B virus data set collected globally during 2009–2014. Australian inter-seasonal sequences are shown in red, seasonal sequences are in blue, and global background sequences are in black, with branch lengths scaled according to the number of nucleotide substitutions per site.
(TXT)

S1 Table GISAID accession numbers and background details for the sequences generated here. (XLSX)
